# The McbR transcription factor links the intracellular folate pool to virulence in enterohemorrhagic *Escherichia coli*

**DOI:** 10.1128/mbio.01016-26

**Published:** 2026-06-09

**Authors:** Quentin Perraud, Vanessa Sperandio

**Affiliations:** 1 Department of Medical Microbiology and Immunology, University of Wisconsin-Madison 5228, Madison, Wisconsin, USA; Massachusetts Institute of Technology, Cambridge, Massachusetts, USA

**Keywords:** enterohemorrhagic *E. coli* (EHEC), type III secretion, virulence regulation, folate metabolism

## Abstract

Bacterial pathogens coordinate the expression of metabolically expensive virulence programs during host colonization. While the impact of environmental cues on virulence has been extensively studied, direct sensing of metabolic intermediates to inform virulence expression is less understood. Competition with the resident microbiota in the case of an infection has a massive impact on the bacteria’s central metabolism, which can be used as a cue for the timely expression of virulence factors. Here, we identify how fluctuations in the intracellular folate pool can affect the expression of virulence factors in the enteric pathogen enterohemorrhagic *E. coli* (EHEC). Using transcriptomics, mutagenesis, and ligand-binding assays, we demonstrate that the EHEC’s type III secretion system, which is a syringe-like apparatus employed by multiple pathogens to infect and manipulate host cell function, is repressed when the bacteria’s folate biosynthetic pathway is pharmacologically disrupted, and that this effect is mediated by two regulatory proteins: PurR and McbR. Additionally, we identify dihydrofolate as a ligand of the previously orphaned McbR transcription regulator. It is notable that folate (vitamin B9) is an essential building block for DNA synthesis, cell formation, and division throughout the tree of life.

**IMPORTANCE:**

Host colonization requires enteric pathogens to tightly regulate their virulence program in response to a multitude of factors. In this work, we show that the bacterial physiological state directly informs pathogenic behavior in enterohemorrhagic *E. coli* (EHEC). Through the study of folate-starved bacterial cultures, we identified dihydrofolate as the endogenous ligand of the McbR transcription factor and demonstrated that McbR is directly linking one-carbon metabolism to virulence gene expression. These findings reveal how metabolites can act as regulatory signals controlling horizontally acquired pathogenicity islands.

## INTRODUCTION

Enterohemorrhagic *Escherichia coli* (EHEC) is a foodborne pathogen causing hemorrhagic colitis in humans, a disease that can lead to hemolytic uremic syndrome (HUS), a life-threatening condition (1). After ingestion by the host, EHEC colonizes the lower intestine, where it adheres to the colonic epithelium, leading to effacement of the microvilli and rearrangement of the cytoskeleton forming pedestal-like structures, known as attaching and effacing (AE) lesions. The majority of the genes responsible for this phenotype are located within a horizontally acquired pathogenicity island, known as the locus of enterocyte effacement (LEE) (2). This pathogenicity island is organized into five major operons (*LEE1–LEE5*) encoding regulators, chaperones, effectors, and a type III secretion system (T3SS). This T3SS is a membrane-spanning assembly of proteins acting as a molecular syringe and allowing the injection of effector proteins directly into the host cell. Among those effectors, the Tir protein is localized on the host cell membrane after injection and allows strong binding of EHEC to the host colonocytes by specifically recognizing the intimin protein on the bacterial surface. As a large multiprotein complex, the T3SS is costly from a metabolic standpoint (3), leading the bacteria to tightly control its expression through the sensing of a wide range of physicochemical cues, including host-derived molecules, nutrients, oxygen, temperature, or quorum sensing molecules. The signal transduction cascades associated with these cues are diverse and can involve two-component systems, effector-binding transcription factors, or even nucleoid structuring protein (4).

These fine-tuned sensing mechanisms offer an opportunity from a drug development standpoint: their disruption could lead to a decrease in EHEC’s ability to colonize its host without necessarily impacting viability at the cellular level. Anti-virulence strategies could represent a major step in slowing the emergence of antibiotic resistance by avoiding strong selective pressure (5). They are also especially significant in the case of EHEC infection: use of traditional antibiotics is known to be a direct cause of HUS through induction of the prophage encoding Shiga toxin (6). This toxin inhibits protein synthesis in the host cells, ultimately causing cell death and leading to vascular injuries and organ damage.

Vitamins are known to be among small molecular cues that modulate EHEC’s virulence (7–9). Among these molecules, tetrahydrofolate (vitamin B9) and its associated pathway in EHEC have not, to our knowledge, been explored as a potential modulator of virulence. Despite folate’s involvement in multiple essential processes (nucleotide biosynthesis, translation initiation, etc.), *E. coli* is unable to import this cofactor from its medium (10). Instead, the bacteria build up their folate pool by biosynthesizing it through the condensation of a pterin, para-aminobenzoate, and glutamate. The pterin precursor of folates is biosynthetized from GTP, while the para-aminobenzoate precursor is synthetized from chorismate. Recent studies suggest that folate impacts virulence in other bacteria (11–13) and describe multiple pharmacological approaches involving sulfonamide drugs as potential therapeutics, which are known to disrupt folate biosynthesis in bacteria (14–16). Given that nutrients such as vitamins can act as virulence modulators in EHEC, that some folate inhibitors are described as modulating virulence in the literature, and the role of folate in virulence regulation of other pathogens, we hypothesized that fluctuations in its intracellular pool of folate in EHEC could influence global transcriptional networks and thereby impact virulence gene expression. This led us to explore the hypothesis that folate biosynthesis itself might be important for the ability of EHEC to express its virulence program.

Here, we show that sub-inhibitory concentrations of the folate biosynthesis inhibitor sulfapyridine repress EHEC’s virulence gene expression through the combined action of PurR and McbR regulators. We also identify dihydrofolate as an endogenous ligand for the McbR transcription factor, providing a new example of coupling between central metabolism and virulence in bacteria.

## RESULTS

### Pharmacological inhibition of folate biosynthesis represses virulence

**Fig 1:**
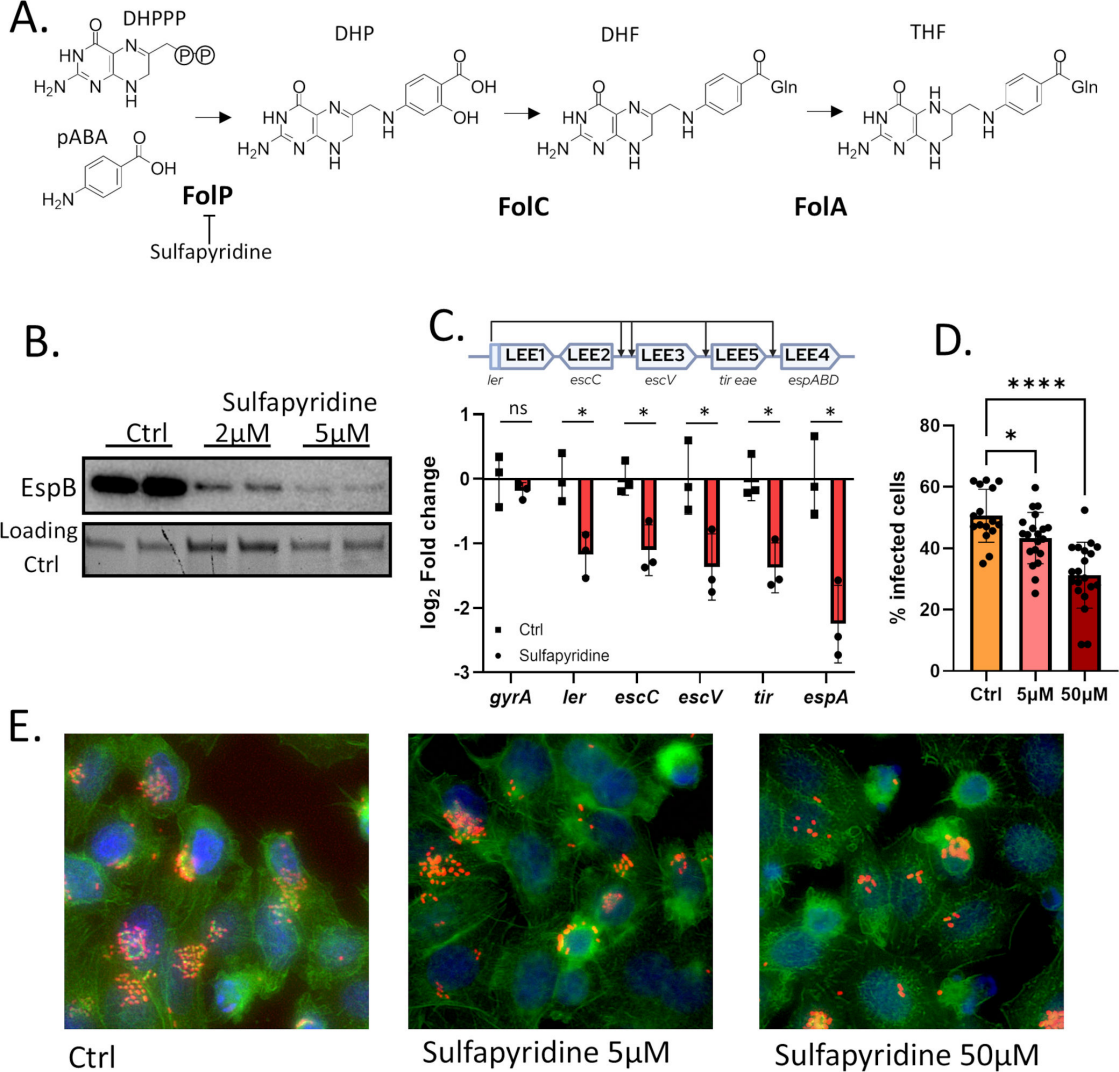
Expression of the LEE pathogenicity island decreases in the presence of sulfapyridine. (**A**) Tetrahydrofolate biosynthesis. 6-hydroxymethyl-7,8-dihydropterin diphosphate (DHPPP) and para-aminobenzoate (pABA) react to form dihydropteroate (DHP) through the catalytic action of the dihydropteroate synthase FolP. This enzyme is the target of sulfonamide drugs, including sulfapyridine. Dihydropteroate is then glutamylated by the dihydrofolate synthetase FolC, forming dihydrofolate (DHF). Dihydrofolate is then reduced to tetrahydrofolate (THF) by the dihydrofolate reductase FolA. (**B**) Western blot demonstrating the effect of sulfapyridine on the secretion of the EspB T3SS translocon protein in standing culture. Bovine serum albumin was spiked in the culture supernatant and used as a loading control to ensure consistency during sample processing. (**C**) RT-qPCR shows that sulfapyridine causes a decrease in the transcription of genes located on each LEE operon without affecting the housekeeping gene *gyrA*. Three biological replicates with three technical replicates each. (**D**) Quantification of the percentage of HeLa cells presenting actin pedestals and attached bacteria after infection with an mCherry-expressing strain of EHEC. (**E**) Representative micrographs obtained after staining of EHEC-mCherry (red)-infected HeLa cells with FITC-phalloidin (actin stain in green) and DAPI (DNA stain). Pedestals are depicted as yellow dots or green underneath red-stained bacteria. Four biological replicates with five technical replicates each. *P < 0.05, **P < 0.01, ***P < 0.001, ****P < 0.0001, ns: nonsignificant. Analysis of variance (ANOVA) test with multiple comparisons.

To probe the role of folate biosynthesis in virulence, we used sulfonamides to pharmacologically inhibit dihydropteroate synthase. This enzyme catalyzes the reaction between para-aminobenzoate and 7,8-dihydropterin-6-yl methyl diphosphate, leading to the formation of dihydropteroate (Fig. 1A). We used sulfapyridine as it is a commercially available chemical analog of an anti-virulence sulfonamide drug that we previously identified in a chemical library screening (17). Sulfapyridine was chosen as a proxy for the previously identified hit because of its structural similarity and belonging to the same class of drug as well as its commercial availability, ensuring a steady supply of quality-controlled molecules.

Evaluation of virulence factor expression in the presence of sulfapyridine on EHEC grown in low-glucose Dulbecco’s modified Eagle medium (DMEM) without shaking revealed a decrease in secretion of the EspB T3SS translocon protein (Fig. 1B), as well as an associated decrease in transcription of LEE-encoded genes (*ler, escC, escV, tir,* and *espA*) across multiple operons (Fig. 1C) at a concentration where it had no impact on bacterial growth (Fig. S1). One consequence of effector injection into host cells by EHEC is a rearrangement of their actin cytoskeleton, leading to the formation of pedestal-like actin structures colocalized with EHEC. Using the method developed by Knutton et al. (18) to visualize these microstructures, we observed a significant marked decrease of EHEC-infected HeLa cells in the presence of sulfapyridine (Fig. 1D and E).

To confirm that LEE repression resulted from inhibition of folate biosynthesis, we performed three complementary experiments: using an inactive prodrug, through chemical rescue, and by using sulfamethoxazole, a different sulfonamide.

**Fig 2:**
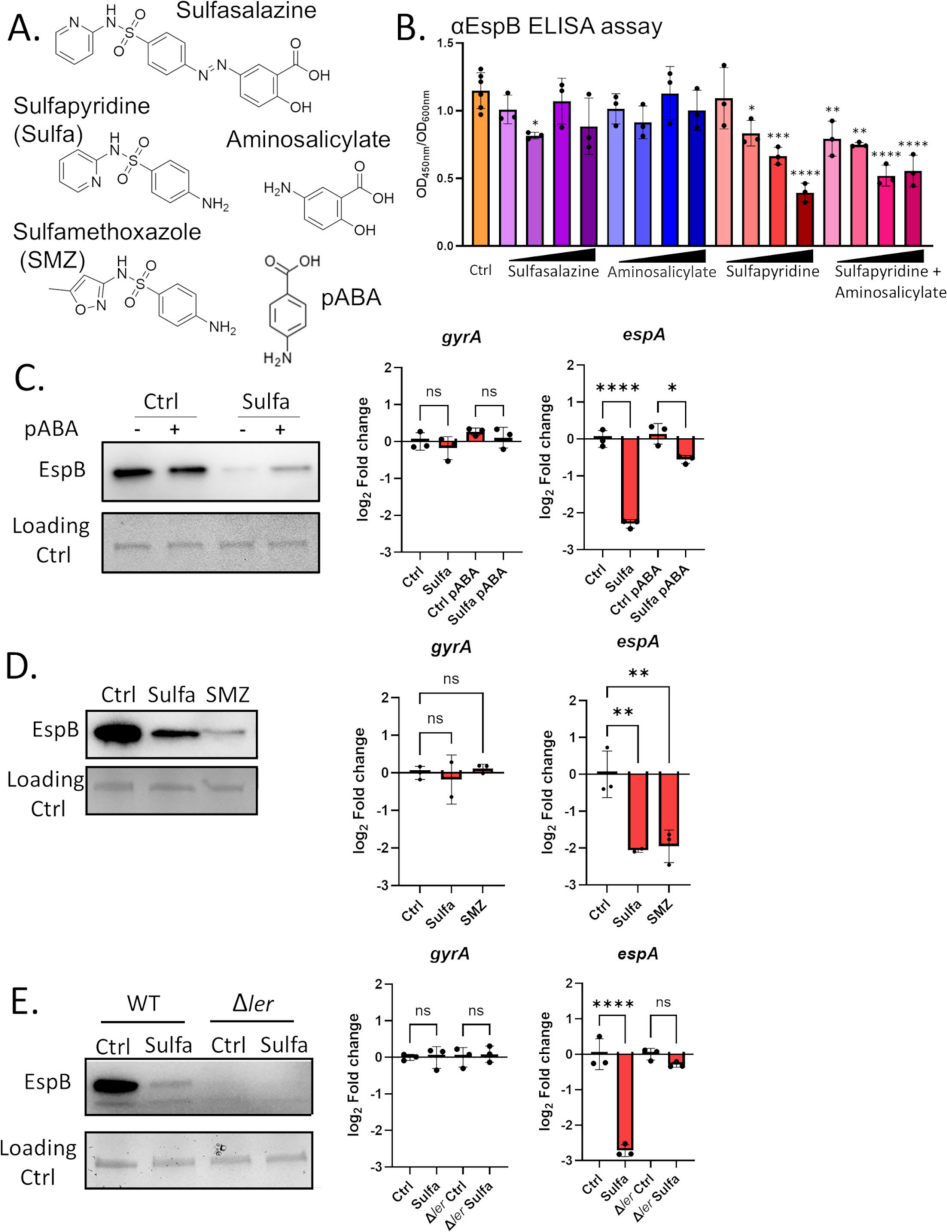
Inhibition of folate biosynthesis represses virulence through the Ler regulator. (**A**) Structures of sulfasalazine, sulfapyridine, 5-aminosalycilate, and sulfamethoxazole. (**B**) Sulfapyridine, but not sulfasalazine, causes a decrease in virulence, as seen with an anti-EspB ELISA. (**C**) RT-qPCR and Western blot show the attenuation of LEE repression caused by the chemical complementation of folate biosynthesis with addition of pABA. (**D**) Sulfamethoxazole, another inhibitor of folate biosynthesis, also causes a decrease in LEE expression, as seen with RT-qPCR and Western blotting. (**E**) RT-qPCR and Western blot demonstrate that the Ler master regulator is necessary for sulfapyridine to have an effect on LEE expression. All RT-qPCR and the ELISAwere performed with three biological replicates averaging three technical replicates each. *P < 0.05, **P < 0.01, ***P < 0.001, ****P < 0.0001, ns: nonsignificant. ANOVA test with multiple comparisons.

We showed that sulfasalazine, a prodrug requiring reduction to release sulfapyridine (19) and 5-aminosalicylate, has no effect on LEE expression (Fig. 2A and B). EHEC is unable to import exogenous folate (10), so to further ensure that this decrease in virulence is due to competitive inhibition of the FolP dihydropteroate synthase (Fig. 1A), we attempted a chemical complementation with para-aminobenzoic acid (pABA), a substrate of FolP that can compete with sulfapyridine for binding to the enzyme. As expected, addition of pABA to sulfapyridine-treated bacteria restored LEE gene transcription near baseline levels and increased EspB secretion in the medium (Fig. 2C). Finally, treatment with another sulfonamide drug, sulfamethoxazole, reproduced the decrease in LEE transcription and EspB secretion (Fig. 2D).

We also showed that the master virulence regulator of the LEE genes Ler, which is encoded within the *LEE1* operon (Fig. 1C) (20), is involved in this effect since sulfapyridine failed to cause any repression of the LEE pathogenicity island when compared to the vehicle control condition in a Δ*ler* background (Fig. 2E). This led us to hypothesize that a transcription factor would modulate *ler* expression when the bacteria is starved for folate.

### The transcription factors PurR and McbR are required for the folate-dependent repression of the LEE

**Fig 3:**
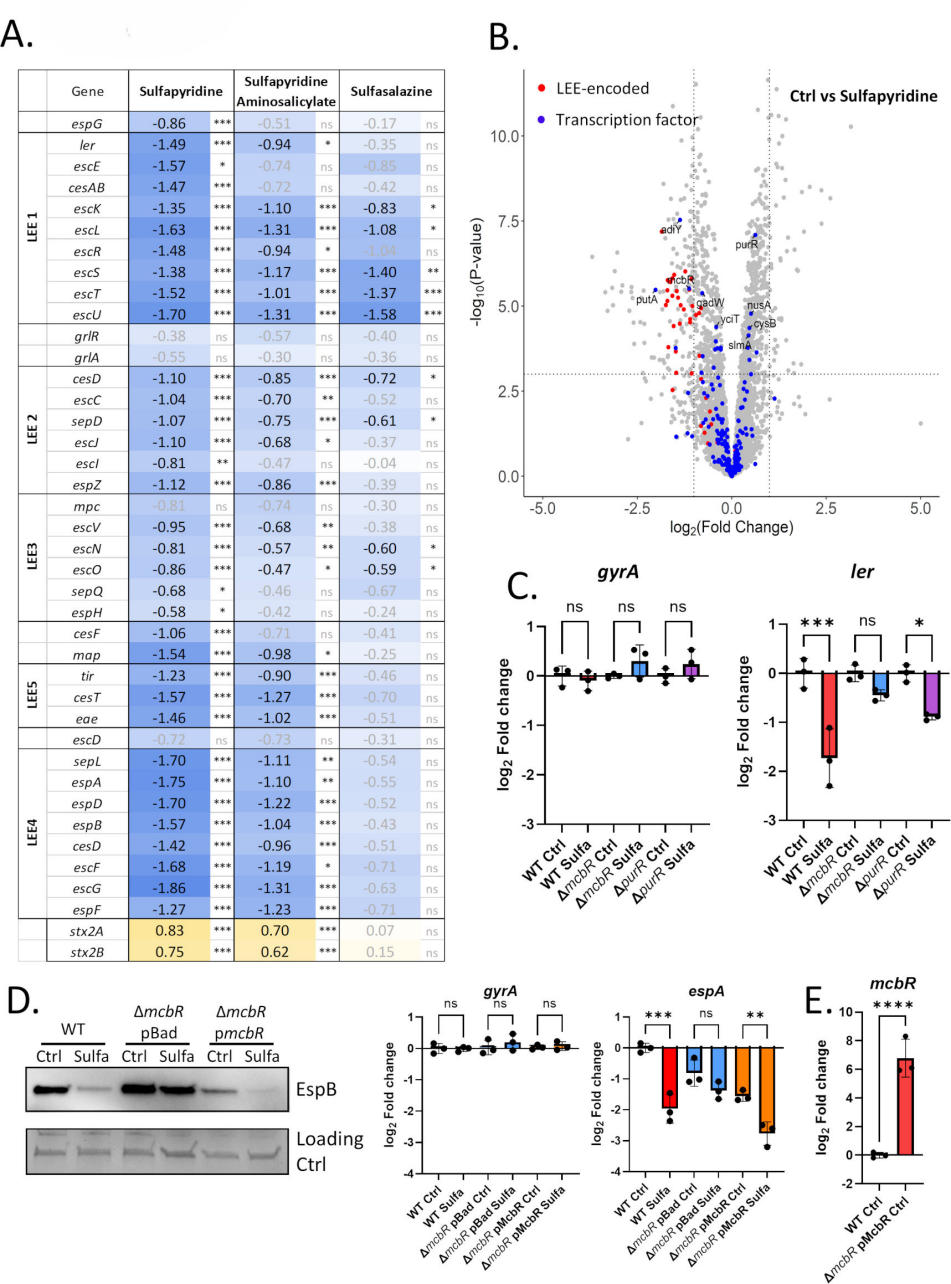
Folate starvation causes transcriptional rewiring, leading the transcription factors PurR and McbR to repress the LEE pathogenicity island. (**A**) Heatmap showing the impact of sulfapyridine, an equimolar mixture of sulfapyridine and 5-aminosalycilate and sulfasalazine on the transcription of the LEE pathogenicity island and Shiga toxin. *P < 0.05, **P < 0.01, ***P < 0.005, ns nonsignificant based on the false discovery rate. (**B**) Differential expression of EHEC transcription factors in the presence of the same molecules. (**C**) Deletion of *mcbR* and *purR* leads to an attenuation of the sulfapyridine effect on the transcriptional regulation of *ler,* as seen with RT-qPCR. (**D**) In *trans* genetic complementation of *mcbR* leads to overexpression of *mcbR* and reveals that McbR acts as a repressor of the LEE, as seen with Western blotting and RT-qPCR. All RT-qPCR assays performed with three biological replicates averaging three technical replicates each. *P < 0.05, **P < 0.01, ***P < 0.001, ****P < 0.0001. ANOVA test with multiple comparisons.

We employed an RNA sequencing approach to confirm our qPCR results obtained with sulfapyridine to generate hypotheses as to what regulatory mechanism might tie the folate pathway to virulence regulation in EHEC. We chose to evaluate the effect of sulfapyridine, of a 1:1 mixture of both 5-aminosalycilate and sulfapyridine, and added sulfasalazine as a negative control since we previously demonstrated that sulfasalazine requires reduction into sulfapyridine and aminosalycilate to be active on EHEC (Fig. 2B). This would allow us to discriminate between a transcriptional response to any xenobiotic and a response specific to folate starvation. We confirmed first that the LEE pathogenicity island was significantly (FDR < 0.05, fold change > 2) downregulated in the presence of sulfapyridine (either alone or in combination with 5-aminosalicylate) while it is not in the presence of sulfasalazine, confirming our initial findings. We also observed that the genes encoding for Shiga-toxin (*stx2a* and *stx2b*) were significantly upregulated in the presence of sulfapyridine, despite using a below-minimum inhibitory concentration (MIC) of the molecule (Fig. 3A), consistent with prior studies reporting Shiga toxin induction in response to sulfonamide stress (6, 21).

A broader investigation into the enrichment of KEGG pathways (Fig. S3) in the presence of sulfapyridine but not sulfasalazine revealed an expected downregulation of the “Pathogenic *Escherichia coli* infection” network (ecs05130), as well as the curli and colanic acid pathway (ecs02026) and quorum sensing (ecs02024). The most upregulated pathway is the ribosome components (ecs03010), followed by fatty acid biosynthesis (ecs00061), biotin metabolism (ecs00780), sulfur metabolism (ecs00920), and purine metabolism (ecs00230).

We evaluated the impact of sulfapyridine on EHEC’s transcription factors, based on their annotation with the GO:0003700 gene ontology term (“DNA-binding transcription factor activity”). We identified a few highly significant genes that were selected for mutagenesis, which are differentially expressed in the presence of sulfapyridine or the 1:1 sulfapyridine:5-aminosalycilate mixture, but not in the presence of sulfasalazine (Fig. 2 and 3B). Among those, *purR* and *mcbR* revealed an attenuation of the effect of sulfapyridine (Fig. 3C), and the mutation of both *purR* and *mcbR* shows a compounding effect compared to the single-mutant strains (Fig. S4). leading us to hypothesize that our molecule might inhibit virulence through those two transcription factors. PurR is a GalR/LacI family transcriptional regulator involved in the repression of purine biosynthesis in the presence of guanine or hypoxanthine (22, 23). PurR has also recently been shown to act as a repressor of the LEE pathogenicity island by directly binding to the promoter of the *ler* gene (24). McbR is a GntR superfamily transcriptional regulator for which the ligand was unknown despite having been crystallized (25). McbR has, to the best of our knowledge, not been linked to virulence regulation in EHEC so far, leading us to pursue a more detailed study of this relationship.

Deletion of *mcbR* alone did not affect LEE gene expression, as assessed with qPCR and Western blotting (Fig. 3D). It did, however, significantly attenuate sulfapyridine-induced LEE repression, confirming our previous result. In *trans* genetic complementation of the deletion mutant led to a very significant overexpression of McbR (Fig. 3E) and a lower expression of LEE genes in our complemented strain when compared to the wild type (whether sulfapyridine-treated or not). Overall, these results led us to hypothesize that McbR functions as a conditional repressor of LEE expression responsive to folate starvation and that the stoichiometry between McbR and its allosteric modulator is important for its activity (Fig. 3D).

### McbR directly binds the *ler* promoter to repress LEE expression, and this activity is allosterically modulated by dihydrofolate

**Fig 4:**
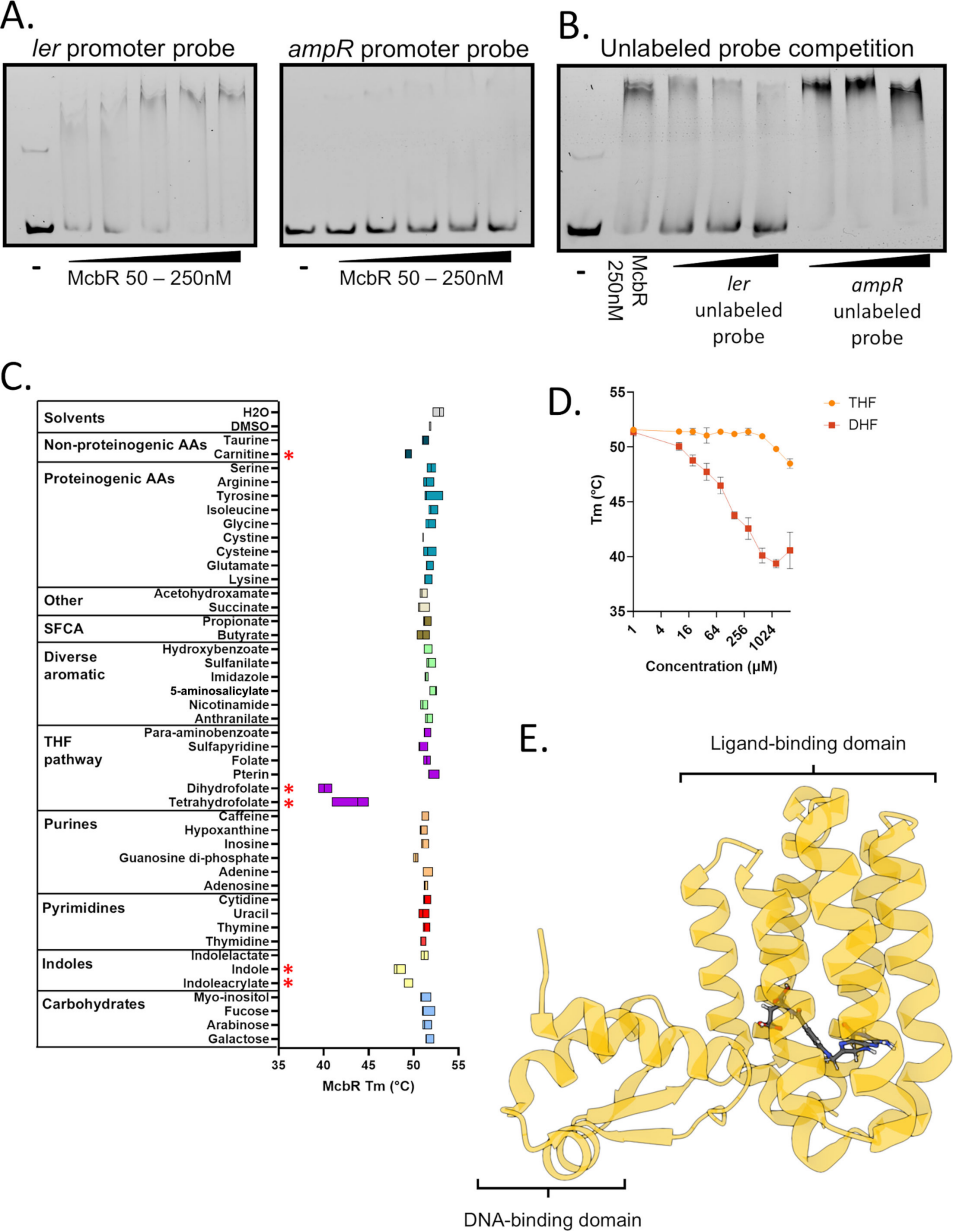
McbR recognizes the *ler* promoter sequence and binds to dihydrofolate. (**A**) EMSA of McbR incubated with a *ler* promoter fluorescent-labeled probe or an *ampR* promoter probe. (**B**) EMSA demonstrating specificity of binding to the *ler* probe by showing that addition of the unlabeled *ler* probe competes with the labeled *ler* probe, while unlabeled non-specific probe does not. (**C**) Result of the thermal shift assay screen performed with high ligand concentration (0.5 mM), identifying tetrahydrofolate and dihydrofolate as potential endogenous ligands for McbR. The result is an average of three independent experiments with three technical replicates each. Significant differences are denoted with a red asterisk (*P* < 0.001), with all other comparisons being nonsignificant compared to the vehicle control. ANOVA test with multiple comparisons. (**D**) Relation between dihydrofolate and tetrahydrofolate concentration and melting temperature of McbR, showing that dihydrofolate’s impact on McbR thermal stability is relevant at lower concentrations compared to tetrahydrofolate. (**E**) Result of the docking of dihydrofolate in the McbR ligand-binding domain using the Attracting Cavity docking engine available on the SwissDock website.

Because the decrease in LEE expression observed with sulfapyridine is dependent on the Ler protein, we hypothesized that McbR might bind to the promoter region of the *ler* gene. Electrophoretic mobility shift assays demonstrated that McbR can bind to the DNA region comprising 169 bp upstream of the *ler* start codon up to 155 bp downstream of the *ler* start codon. The same concentration of McbR was unable to bind to a control DNA fragment (Fig. 4A). Specificity of the binding was further confirmed through an unlabeled probe competition assay: excess of unlabeled *ler* probe led to abrogation of the shift, while addition of an unrelated competitor did not (Fig. 4B).

Because McbR belongs to the GntR superfamily of transcription factors, we assumed that ligand-binding would modulate McbR’s DNA binding activity and hypothesized that the intracellular pool of the endogenous McbR ligand would shift in the presence of sulfapyridine (26). To investigate this, we used a high-throughput protein thermal shift assay and screened for ligands able to affect the thermal stability of McbR. We identified both tetrahydrofolate itself and dihydrofolate as strongly negatively impacting the thermal stability of our purified protein (Δ*T* ≈ 8.6°C and Δ*T* ≈ 11.7°C, respectively, at a concentration of 0.5 mM of ligand for 1 µM of protein) (Fig. 4C). Specificity was confirmed by running the screening on the control protein supplied with the thermal shift assay kit (Fig. S5). Those two hit compounds were then further evaluated with varying concentrations of ligand, leading us to identify dihydrofolate as the ligand with higher affinity for McbR (Fig. 4D). This result was congruent with an *in silico* docking of dihydrofolate in the McbR ligand-binding cavity identified by Lord et al. (25) (Fig. 4E; Fig. S6).

## DISCUSSION

Bacterial pathogens must engage in a complex balancing act, coordinating the expression of energetically costly virulence programs with their metabolic status to successfully colonize their host (3, 27, 28). Environmental factors regulating virulence have been studied extensively in enteric bacteria since the inception of microbial pathogenesis, but cross-regulation mechanisms between the metabolic state of the bacteria and virulence gene expression remain incompletely understood. Here, we identify a folate-dependent regulatory network linking the one-carbon metabolism with regulation of the LEE pathogenicity island in EHEC. We show that disruption of folate biosynthesis leads to a decrease in virulence mediated by the transcription factors PurR and McbR. Furthermore, we identify dihydrofolate as the endogenous ligand for the McbR protein, negatively affecting its ability to repress virulence. These findings highlight previously unknown mechanisms through which fluctuations in the cellular folate pool can lead to differential gene expression.

The PurR derepression that we observed when the bacteria are starved for folate can be explained in the broader context of one-carbon metabolism. *De novo* biosynthesis of purines requires the cofactor 10-formyltetrahydrofolate to be present in concentrations high enough for the PurH enzyme to formylate the AICAR intermediate, closing the six-membered ring of purines (29). Additionally, biosynthesis of tetrahydrofolate itself requires GTP as a starting point for the formation of pterin (30). These two links between purine and one-carbon metabolism allow us to assume that the presence of a folate biosynthesis inhibitor is inevitably going to lead to a change in purine flux. Besides the already mentioned role of PurR in virulence regulation (24), purine availability has been shown to be an important driver in cross-species interactions shaping virulence factor expression in EHEC (31).

McbR is a regulator that has been first described as involved in stress responses, drug tolerance, colanic acid production, and biofilm formation (32, 33). One of these earlier reports suggests that McbR might cross-regulate H-NS-silenced genes (34). H-NS is known as a “cell immunity” protein that silences horizontally acquired DNA, such as the LEE pathogenicity island. ChIP-seq experiments on *E. coli* K12 provide insights to the binding site of the K12 McbR, which seem to include an ATATAT motif and bind to a very large number of AT-rich sites in the genome (35). Of note, the LEE is extremely AT-rich, having a GC content of 34% in contrast to the 50% GC content of the ancestral *E. coli* genome (36).

**Fig 5:**
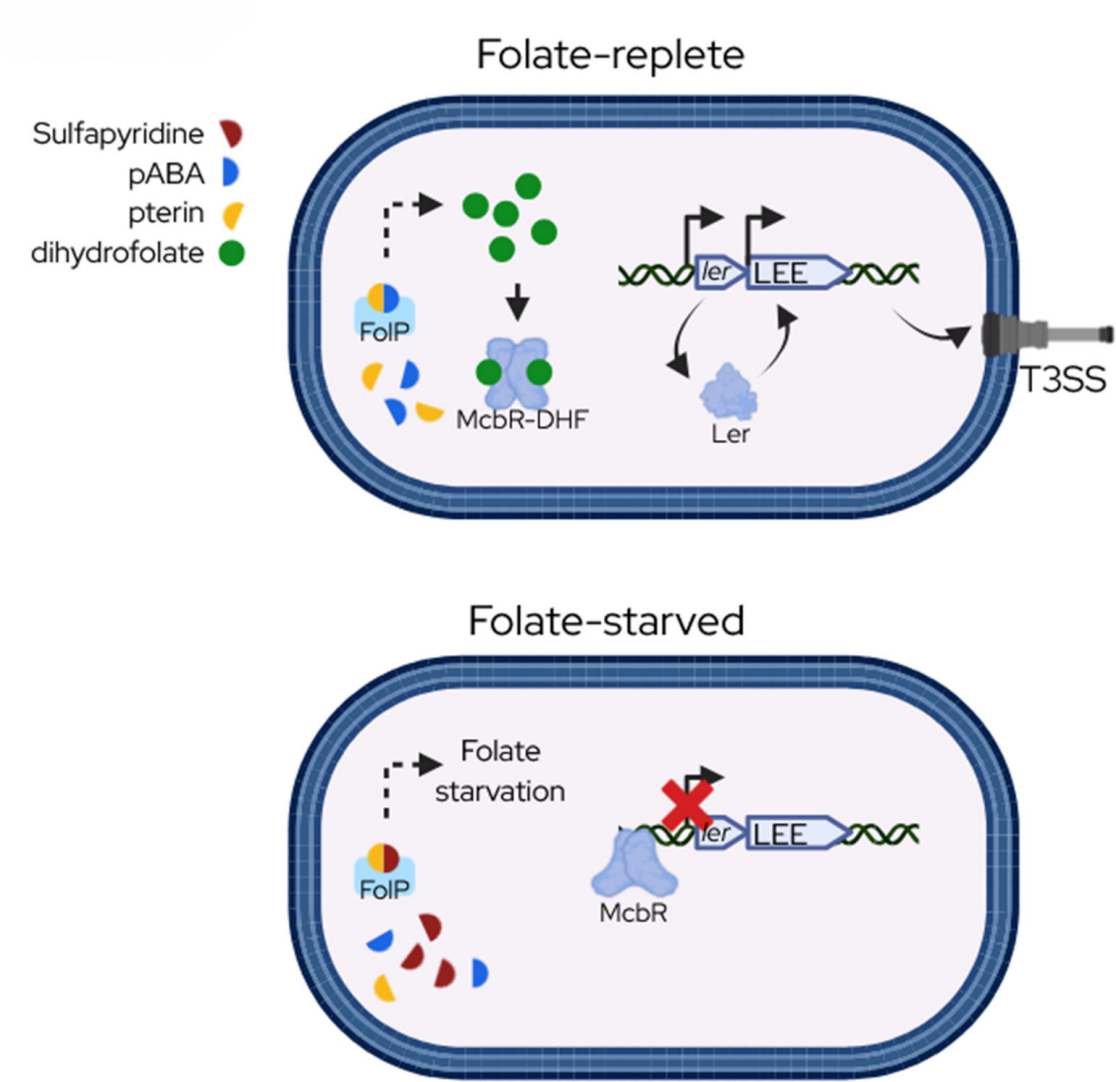
Model of the mechanism through which depletion of intracellular folate pool leads to virulence decrease through the action of McbR. In homeostatic conditions, the dihydropteroate synthase FolP catalyzes the synthesis of dihydropteroate from hydroxymethyl-7,8-dihydropterin pyrophosphate and pABA, further leading to biosynthesis of dihydrofolate and tetrahydrofolate. Dihydrofolate is then binding to McbR, leading to a conformational shift and a loss of affinity for its binding site on the promoter sequence of *ler*. Ler is then able to activate the expression of the LEE pathogenicity island in response to extracellular signals, and the bacteria express its T3SS. In the presence of a dihydropteroate synthase inhibitor, depletion of the intracellular folate pool leads to McbR being present in its active form, where it can bind to the *ler* promoter sequence and stops production of T3SS in response to this metabolic stress.

McbR belongs to the VanR sub-family of the GntR superfamily of transcription regulators. This class of transcription factors are composed of a helix-turn-helix DNA-binding domain, an effector-binding domain composed of six anti-parallel helices and a dimerization domain (26). In the case of FadR, the best studied GntR superfamily regulator, binding of the myristoyl-CoA effector causes a physical separation of the two DNA-binding domains, leading to the release of the protein from its target sequence (26). A similar mechanism would be congruent with our genetic complementation experiment: at baseline, McbR bound to dihydrofolate would not exert any effect on the McbR regulon; however, we propose that disruption of this biosynthesis pathway with sulfa drug would lead to DHF becoming less available and causing the binding of McbR dimers in the promoter region of the *ler* master regulator (Fig. 5). In the case of our complemented mutant strain overexpressing McbR compared to homeostatic conditions, we could see a baseline decrease in virulence, which could be explained by the too high concentration of McbR in the cell for the amount of DHF present to bind to the *ler* promoter. The identification of dihydrofolate as the endogenous ligand of McbR opens the door to a better characterization of this transcription factor, the investigation of its full regulon in folate-starved and replete conditions, as well as investigating a potential role in one-carbon metabolism regulation. This is the first description of a transcription factor as a folate sensor in gamma-proteobacteria. This is in stark contrast to *Bacillota*, where folates are sensed by riboswitches controlling the expression of folate biosynthesis or transport genes (37, 38).

While dihydrofolate binds to McbR (Fig. 4C and D), the question remains of whether this molecule is the only McbR ligand *in vivo*. The folate biosynthesis pathway is complex, involving many steps, intermediates, and minor side products (39) that might also bind to McbR. Addition of sulfapyridine itself can also lead to formation of new products: it has been described that dihydropteroate synthase can catalyze the formation of a pterin-sulfonamide adduct, thereby depleting the pterin pool in the cell (40, 41). These molecules are structural analogs of dihydrofolate and tetrahydrofolate, and their binding activity to McbR should be assessed in the future.

The crystal structure of McbR purified by Lord et al. did not allow them to identify a co-crystalized ligand corresponding to the electron density that they observe in the binding domain of McbR (25). We could see that dihydrofolate does not fit that electron density either, which was not surprising considering the result we obtained over the course of our complementation experiment: we could see that even a mild overexpression of McbR was enough to strongly affect its activity by shifting the ratio of McbR and its ligand. We can assume that an overexpression of McbR strong enough to purify it and obtain protein crystals would surely amplify that phenomenon and that there would not be enough endogenous ligands to bind to the large amounts of McbR protein in the cell. We can also question how a unique property of folates fits into this model: their ability to be polyglutamylated. Our experiments involved mono-glutamylated dihydrofolate, and our *in silico* docking study would suggest that polyglutamylation of dihydrofolate would cause a loss of binding affinity to McbR due to steric hindrance (Fig. 4E).

In summary, our study reveals that perturbations of the folate biosynthesis pathway in EHEC influences the expression of the LEE through a regulatory network involving the PurR and McbR proteins. The coupling of central metabolism and virulence that we observed adds to a growing body of examples of metabolites being used as a source of regulatory information in multiple bacteria (27, 42). Metabolic transcription factors have been shown to be used as virulence regulators (43, 44), allowing them to tie the expression of costly virulence programs to the metabolic status. This also integrates the expression of horizontally acquired genes to the ancestral bacterial chromosome. Our identification of McbR as a folate sensor supports a model in which fluctuations in the one-carbon metabolism could be used as a complementary read-out to extracellular cues, resulting in a multilayered decision-making system for the expression of virulence factors. The presence of the McbR transcription factor in multiple pathogenic bacteria (*Salmonella*, *Shigella*, *Enterobacter*, *Bordetella*, *Burkholderia*, etc.) begs the question of whether a similar role of McbR in virulence regulation could be demonstrated for those organisms and more broadly what global transcriptional responses could be induced by the sensing of folate starvation by McbR.

## MATERIALS AND METHODS

### Strains, plasmid, growth conditions, and chemicals

*Escherichia coli* strain 86-24 (45) was routinely grown in LB medium at 37**°**C. For *in vitro* assays, bacteria were grown in Dulbecco’s modified Eagle medium (DMEM) with low glucose (1 g/L) to late-log phase in standing cultures at 37**°**C with 5% CO_2._ HeLa cells were maintained at 37**°**C with 5% CO_2_ in DMEM with glucose (4.5 g/L), penicillin (100 U/mL), streptomycin (100 µg/mL), and 10% heat-inactivated fetal bovine serum. Sulfapyridine, 5-aminosalycilic acid, sulfasalazine, and tetrahydrofolate were sourced from Cayman Chemical. Dihydrofolate was sourced from Sigma. The cell culture-grade, anhydrous dimethyl sulfoxide used as a vehicle for *in vitro* assay was sourced from ThermoFisher.

### Growth assay

Bacterial growth kinetics were obtained using a Tecan Spark plate reader, supplemented with plating and CFU counting when appropriate.

### Deletion mutants

Deletion mutant construction in EHEC was carried out using the lambda red recombineering technique (46). Briefly, PCR products containing chloramphenicol or kanamycin resistance cassettes flanked by 60-bp homologous sequences to the targeted gene were transformed into EHEC expressing the *exo*, *gam,* and *beta* genes carried on a pKD46 plasmid. After transformation, recombinant bacteria were selected on chloramphenicol or kanamycin LB plates, and replacement of the target gene with the antibiotic cassette was ensured with PCR and/or whole-genome sequencing.

### RNA extraction and RT-qPCR

Bacterial cultures were grown standing in DMEM (1 g/L glucose) to mid-log phase and harvested by centrifugation before being resuspended in 1 mL of TRIzol reagent (Invitrogen) and kept at −80°C until extraction. The extraction was performed following the manufacturer’s protocol. Briefly, the TRIzol was mixed with chloroform before being centrifuged. The RNA-containing fraction was then precipitated with isopropanol and the resulting pellet washed with a 75% solution of ethanol. After resuspension in RNase-free water (Ambion), DNase digestion was performed using the Turbo DNA-free kit (Invitrogen) following the manufacturer’s instructions. The resulting solution was then cleaned up using the RNA Clean XP magnetic bead solution (Beckman Coulter). RNA concentration was measured with a DeNovix DS-11 Spectrophotometer, and cDNA synthesis was performed on 2 µg of RNA using the SuperScript IV reverse transcriptase (ThermoFisher). The cDNAs were mixed with an appropriate quantity of primers and SybrGreen (Applied Biosystems), and quantitative PCR was performed using a Quantstudio 6 Flex (Applied Biosystems) thermocycler. Data were normalized to the *rpoA* housekeeping gene using the ΔΔCt method.

### ELISA

Bacteria were seeded at 1/100 dilution in DMEM with low glucose (1 g/L) in 96-well plates. An EHEC Δ*espB* strain was used as a negative control, and 6His-tagged purified EspB was used as a positive control. After 5 h of growth at 37**°**C with 5% CO_2_, absorbance at 600 nm was measured with a Spark plate reader (Tecan), and cultures were quenched with sodium azide. The spent medium was harvested by centrifugation, buffered to pH 8.2 with carbonate/bicarbonate, and mixed with protease inhibitor cocktail (Sigma). The supernatant was then added to a MaxiSorp ELISA plate (Nunc) and incubated overnight for protein binding. The plate was then incubated with 5% milk in PBST, washed, incubated with an anti-EspB rabbit antibody, washed again, and finally incubated with an HRP-conjugated anti-rabbit goat antibody. Plates were developed with the colorimetric reagent 3,3′,5,5′-tetramethylbenzidine (Sigma), and reactions were quenched with 1 N HCl after blue color started to appear in the plate wells. Absorbance at 450 nm was then measured with a plate reader, and the ratio of *A*_450_/*A*_600_ was used to evaluate EspB secretion in culture supernatants.

### Western blotting

Bacterial cultures were grown standing in DMEM (1 g/L glucose) to mid-log phase. For analysis on secreted proteins, the cultures were centrifugated, bovine serum albumin at 5 µg/mL was added as a loading control, and protease inhibitor cocktail at 1 µL/mL (Sigma) was added to minimize protein loss. The protein-containing supernatant was then sterile filtered with Steriflip 0.22-µm filters (Millipore) and concentrated using an Amicon ultracentrifugation system with a 10-kDa cutoff (Millipore). Concentrated samples were mixed with the loading dye, boiled, run on pre-cast MiniProteans TGX gels (Biorad) and imaged using a Gel Doc (Biorad). Proteins were transferred onto PVDF membranes using a TransBlot semi-dry blotting system. Membranes were blocked with 5% milk in PBST buffer, probed with anti-EspB rabbit antibodies, washed and probed with an HRP-conjugated goat antibody, washed, developed with SuperSignal West Femto ECL reagent (ThermoFisher), and finally imaged using a Gel Doc (Biorad). For analysis of intracellular proteins, the equivalent of 0.5 mL of the culture at OD_600_ of 1 was harvested by centrifugation and resuspended in 500 µL of lysis buffer. The same Western blotting workflow was used as for secreted protein analysis.

### Fluorescent actin staining

The method described in Knutton et al. (18) was used to evaluate actin pedestal formation. HeLa cells were seeded at 80% confluency on glass coverslips in 12-well cell culture plates and allowed to attach overnight. The cells were then incubated with EHEC carrying pcDNA3.1-mCherry at a multiplicity of infection of 100 for 2 h, then washed delicately with PBS, and incubated for 2 h more at 37**°**C with 5% CO_2_. The cells were then washed with PBS, fixed with 2% formaldehyde, and permeabilized with 0.2% Triton X100. The coverslips were then stained with a 1 µg/mL solution of fluorescein isothiocyanate-conjugated phalloidin and mounted using the ProLong Diamond anti-fade with DAPI reagent (Invitrogen). The slides were allowed to cure overnight and were then blinded and imaged with a Revolve microscope (Echo).

### RNA sequencing

RNAs from five biological replicates were extracted using the previously described method and sent to SeqCenter LLC for sequencing. The Stranded Total RNA Prep, Ligation with Ribo-Zero Plus kit (Illumina) was used to prepare RNA libraries. Sequencing was carried out on a NovaSeq X Plus instrument (Illumina), resulting in 150-bp paired-end reads. The bcl-convert (v4.1.5) app was used for quality control and adapter trimming and demultiplexing. Transcript quantification and differential expression analysis were performed using the *E. coli* O157:H7 strain Sakai reference.

### Protein purification

*mcbR* was inserted in the multiple cloning site of the pET-28a + plasmid using HiFi DNA Assembly (NEB) before being transformed in *E. coli* BL21(DE3). Expression of C-terminal-tagged McbR-6His was induced with 1 mM IPTG before cells were harvested and kept at −80°C until further processing. The bacteria were lyzed using a sonicator (QSonica) and the recombinant protein purified on a HisTrap HF Ni-NTA chromatography column (Cytiva) adapted to an AKTA Start FPLC system (GE) following the manufacturer’s instructions. The fraction containing the recombinant protein was dialyzed overnight in order to remove excess imidazole, and the concentration was evaluated using a Qubit fluorimeter with the protein BR assay kit (ThermoFisher).

### Electrophoretic mobility shift assay

Fluorescent probes were obtained by PCR using either genomic DNA or plasmid and 6-FAM-labeled primers. The probes were then purified by electrophoresis followed by gel extraction (Qiagen Gel extraction kit). Reactions (20 µL) were set up including 4 µL of 5× buffer (50 mM Tris-HCl, pH 7.5, 25 mM NaCl, 250 µg/mL BSA, 5 mM EDTA, and 250 mM KCl), 1.5 µL of poly-dIdC non-specific competitor (500 ng/µL), 2 µL of 50% glycerol, 80 fmol of 6-FAM-labeled probe, as well as purified McbR and unlabeled probes when appropriate. 30 min of incubation, the binding reactions were resolved on non-denaturing TBE polyacrylamide gels in 0.5× Tris–borate–EDTA and imaged using a Typhoon FLA 9000 (Amersham).

### Protein thermal shift assay

The protein thermal shift assays were set up following the recommendations from the Protein Thermal Shift Kit (Applied Biosystems). Purified protein at a final concentration of 2 µM was incubated at room temperature for 30 min with 0.5 mM of ligand (for initial screening) or varying concentrations (for further characterization) in 100 mM KPO_4_ (pH 7) buffer. After incubation, protein thermal shift dye was added to a final concentration of 1× and incubated for an extra 10 min. Reactions without the ligand, without protein, and without dye were added as control, as well as reaction using the protein provided in the Protein Thermal Shift Starter Kit to check for specificity to McbR. The reactions were analyzed using a Quantstudio 6 Flex thermocycler (Applied Biosystems) following a temperature gradient from 25°C to 99°C at a rate of 0.05°C/s, with excitation wavelength set to 520 ± 10 nm and emission wavelength set to 558 ± 11 nm. The raw fluorescence data were processed using the wTSA-CRAFT (47, 48) server to compute the melting temperature for each reaction.

## Data Availability

The RNA sequencing results and raw data are available for download on the GEO database (accession number GSE322789).

## SUPPLEMENTAL MATERIAL

Supplemental material mbio.01016-26-s0001.pdf Supplemental tables and figures.

ASM does not own the copyrights to Supplemental Material that may be linked to, or accessed through, an article. The authors have granted ASM a non-exclusive, world-wide license to publish the Supplemental Material files. Please contact the corresponding author directly for reuse.
