# Supplementary material for: The McbR transcription factor links the intracellular folate pool to virulence in enterohemorrhagic Escherichia coli
Source: mBio. 2026 Jun 9;17(7):e01016-26. doi: 10.1128/mbio.01016-26 (PMC13343897; doi:10.1128/mbio.01016-26)
Supplement: Supplemental material — Supplemental tables and figures. [file mbio.01016-26-s0001.pdf]

Table S1. Strains

Strain	Background	Purpose	Reference
EHEC WT	*E. coli* O157:H7 str. 86-24 (Strep^{R})	Wild type strain	Griffin *et al.*, 1988
Δ*ler*	*E. coli* O157:H7 str. 86-24 (Strep^{R})	*Ler* deletion mutant	Elliott *et al.*, 2000
Δ*purR*	*E. coli* O157:H7 str. 86-24 (Strep^{R} Cm^{R})	*PurR* deletion mutant	This study
Δ*mcbR*	*E. coli* O157:H7 str. 86-24 (Strep^{R} Cm^{R})	*McbR* deletion mutant	This study
Δ*purR* Δ*mcbR*	*E. coli* O157:H7 str. 86-24 (Strep^{R} Cm^{R} Kan^{R})	*McbR PurR* double mutant	This study
Δ*mcbR* pBADmycHis	*E. coli* O157:H7 str. 86-24 (Strep^{R} Cm^{R}Amp^{R})	*McbR* mutant with empty plasmid	This study
Δ*mcbR* pMcbR	*E. coli* O157:H7 str. 86-24 (Strep^{R} Cm^{R}Amp^{R})	*McbR* mutant with complementation plasmid	This study

Table S2. Plasmids

Plasmid	Resistance	Purpose	Reference
pBADmycHis	Amp^{R}	Used as a control	Invitrogen
pMcbR	Amp^{R}	Used as a complementation vector (without induction)	This study
pET28-mcbR	Amp^{R}	Expression vector for purification of recombinant McbR-6His	This study
pKD46	Amp^{R}	Plasmid carrying Lambda red machinery	Datsenko and Wanner 2000
pKD4	Amp^{R} Kan^{R}	Plasmid carrying kanamycine cassette for lambda red recombineering	Datsenko and Wanner 2000

pKD3	Amp^{R} Cm^{R}	Plasmid carrying chloramphenicol cassette for lambda red recombineering	Datsenko and Wanner 2000
pDP151	Amp^{R}	mCherry expression plasmid	

Table S3. Primers

Primer	Sequence	Purpose
Ler1-169-6FAM-F	/6-FAM/GTTGACATTTAATGATAATGTATTTTACACATTAG	EMSA
Ler1-+155-6FAM-R	/6-FAM/CTCAATTACACTTTGAACTTCCTGCTCTCG	EMSA
Amp-6FAM-F	/6-FAM/GCTGAAGATCAGTTGGGTGCA	EMSA
Amp-6FAM-R	/6-FAM/CTGAGAATAGTGTATGCGGC	EMSA
mcbR-LR-F	CAGGTGAATTCCTTCTGCCATGCAGGCAGGGTTGGACAGAAAACACGCGAAGGAATTAAAGTGTAGGCTGGAGCTGCTTCG	Mutagenesis
mcbR-LR-R	GAATATCGCATGGCGATTTGAGTAATTACCTTGATGCCCGGTATTTGCCGGGCATTTACTCATATGAATATCCTCCTTAG	Mutagenesis
purR-LR-F	AGGCAAACGTTTACCTTGCGATTTTGCAGGAGCTGAAGTTAGGGTCTGGAGTGAAATGGAGTGTAGGCTGGAGCTGCTTCG	Mutagenesis
purR-LR-R	TAGTGAGACGCTGAATAAGGAGTGGCTGCGGGAGCCGGAAGAGACTCCCGCAACGGGTGACATATGAATATCCTCCTTAG	Mutagenesis
qPCR-gyrA-F	TGGTGACTCGGCAGTTTATG	RT-qPCR
qPCR-gyrA-R	CGTCGATGGAACCGAAGTTA	RT-qPCR
qPCR-mcbR-F	TACAGCAGGCGCAAGAAA	RT-qPCR
qPCR-mcbR-R	CTCACACAGGATGGGCATATT	RT-qPCR
qPCR-purR-F	GCGTGGAACAATCTTGAGAAAC	RT-qPCR
qPCR-purR-R	CTGGGTACTCAGAACACATCAC	RT-qPCR

qPCR-rpoA-F	GTGACCCTTGAGCCTTTAGAG	RT-qPCR
qPCR-rpoA-R	ACACCATCAATCTCAACCTCG	RT-qPCR
qPCR-ler-F	CGAGAGCAGGAAGTTCAAAGTG	RT-qPCR
qPCR-ler-R	ACACCTTTCGAATGAGTTCCG	RT-qPCR
qPCR-escC-F	CTGAAGACAATGGCAAGTAATGG	RT-qPCR
qPCR-escC-R	ACTGCATTAAGACGTGGATCAG	RT-qPCR
qPCR-escV-F	GAGTGCAAAAGGAAAGCCAG	RT-qPCR
qPCR-escV-R	ATGATACCAGCAATAGCGTC	RT-qPCR
qPCR-tir-F	GAGGGAGTCAAATAGCGGTG	RT-qPCR
qPCR-tir-R	ATCTGAACGAAGGCTGGAAG	RT-qPCR
qPCR-espA-F	AGCTATTTGAGGAACTCGGTG	RT-qPCR
qPCR-espA-R	CATCTTTTGTGCCGTGGTTG	RT-qPCR
pBad-f	AAGCTTGGGCCCGAACA	Complementation
pBad-r	CGAGCTCGGATCCATGGT	Complementation
mcbR-f-cmp	TTTGGGCTAACAGGAGGAATTAACCATGGATCCGAG CTCGCGAAGGAATTAAAatgCCTG	Complementation
mcbR-r-cmp	GATCCTCTTCTGAGATGAGTTTTTGTTCGGGCCCAAG CTTACGATTGTATTGCTGGTATAAA	Complementation
mcbR-28-F	TAAGAAGGAGATATACCATGCCTGGAACGGAAAAAA TG	Expression vector
pET28-mcb-R	TTCATTTTTTCCGTTCCAGGCATGGTATATCTCCTTCT TAAAG	Expression vector
mcbR-28-R	TTGTCGACGGAGCTCGAATTACGATTGTATTGCTGGT	Expression vector
pET28-mcb-F	TATACCAGCAATACAATCGTAATTCGAGCTCCGTC	Expression vector

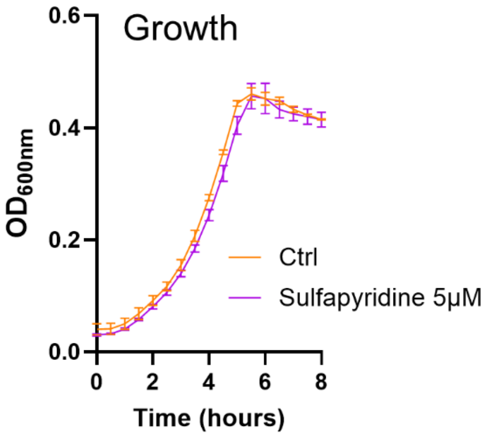

Figure S1. Growth of EHEC in presence or absence of sulfapyridine shows no viability impact at the concentration used for RT-qPCR and Western blotting analysis.

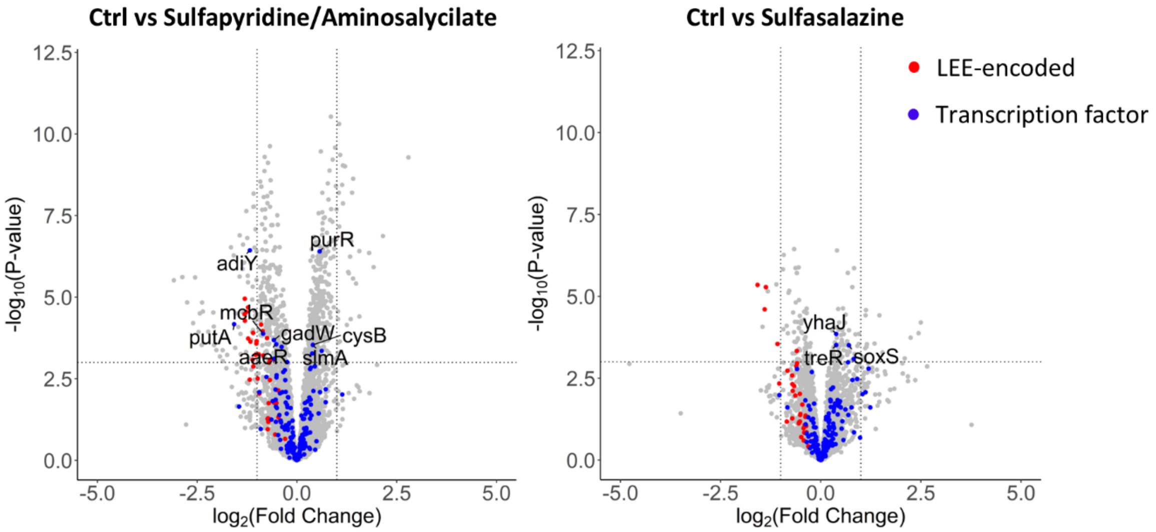

Figure S2. Volcano plot of the differential RNAseq experiment for the comparisons Ctrl vs Sulfapyridine/Aminosalycilate and Ctrl vs Sulfasalazine.

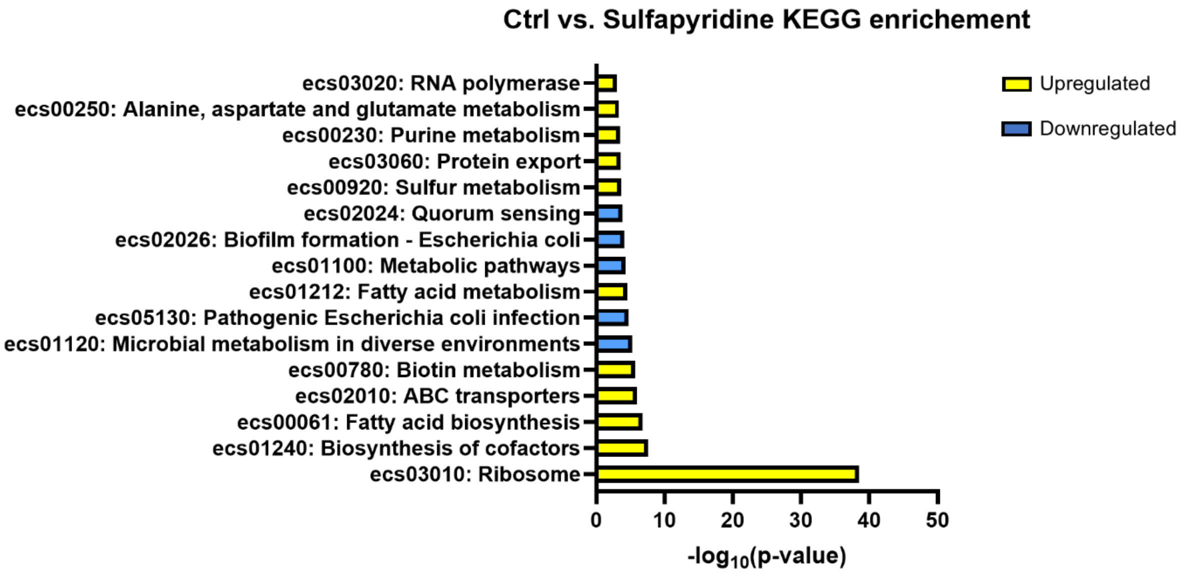

Figure S3. KEGG enrichment analysis for the vehicle control versus sulfapyridine treated condition. Only pathway with p<0.001 are displayed.

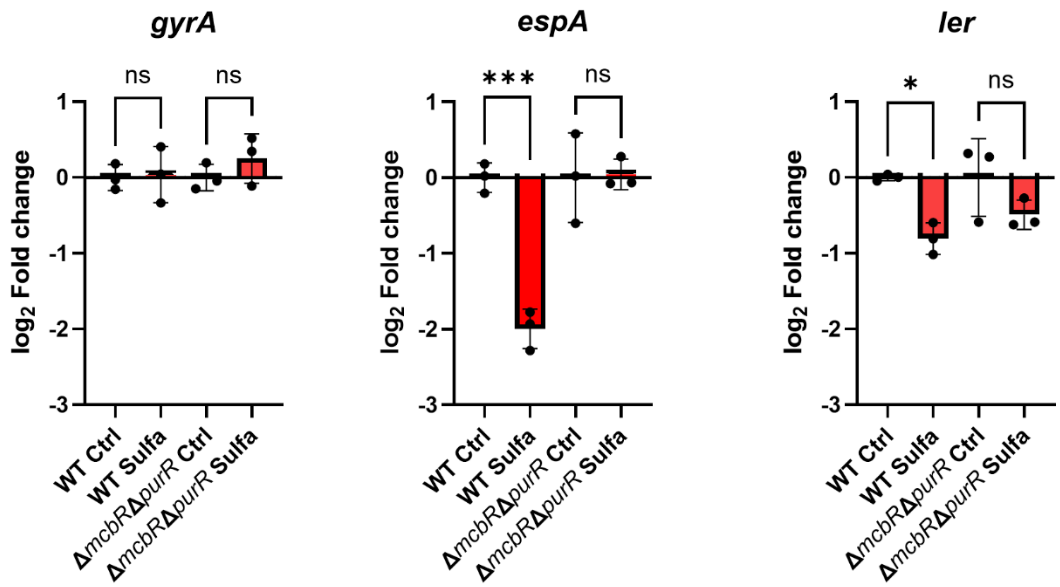

Figure S4. RT-qPCR analysis of sulfapyridine effect on LEE genes in a Δ*mcbR* Δ*purR* double mutant strain. Three biological replicates with three technical replicates each, *p>0.01 *** p<0.001 ns non-significant. ANOVA test with multiple comparisons

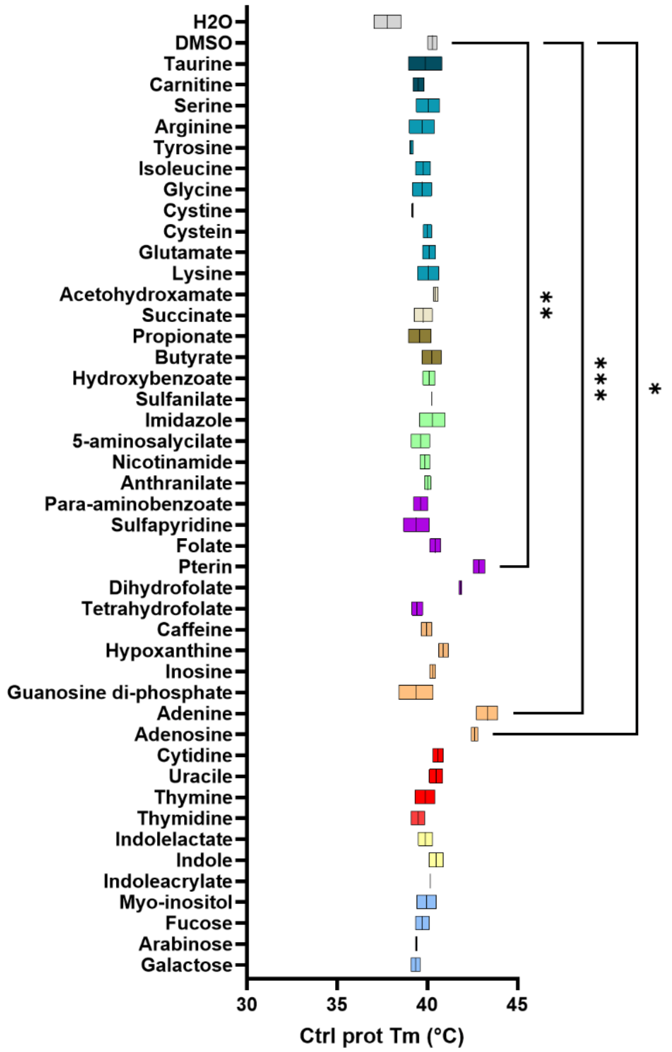

Figure S5. Protein thermal shift screening using the control protein supplied in the Protein Thermal Shift™ Starter Kit (ThermoFisher) instead to McbR to control for non-specific impact of ligand on protein melting temperature. Two independent experiments with three technical replicates each, *p>0.01 **p<0.05 *** p<0.001. ANOVA test with multiple comparisons.

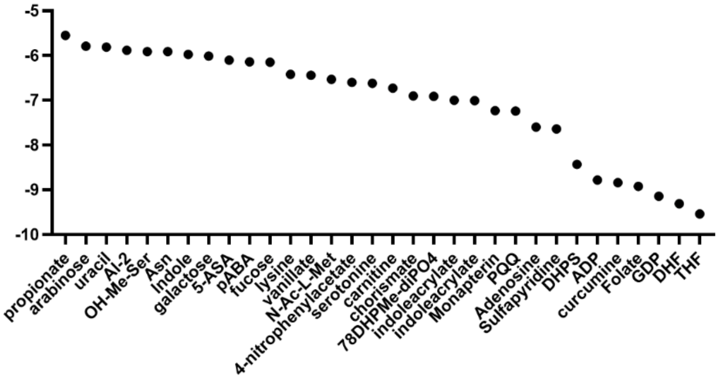

Figure S6. ΔG^0 values obtained after docking of various structures in the 4P9F PDB structure of McbR. The compounds were either randomly selected, informed by RNAseq results or by genetic proximity of annotated genes to *mcbR*. The Attracting Cavity docking engine available on the SwissDock website was used to carry out the analysis[49, 50].
